# Quantification of the prevalence of harms in healthcare related to drug treatment: reflections regarding the use of definitions developed for other settings to estimate the magnitude of the problem

**DOI:** 10.1007/s00228-024-03766-7

**Published:** 2024-10-17

**Authors:** Susanna M. Wallerstedt, Mikael Hoffmann

**Affiliations:** 1 Department of Pharmacology, Sahlgrenska Academy, University of Gothenburg, Box 431, SE-405 30 Gothenburg, Sweden; 2 HTA-Centrum, Sahlgrenska University Hospital, Gothenburg, Sweden; 3 The NEPI Foundation – Swedish Network for Pharmacoepidemiology, Linköping, Sweden; 4 Department of Health, Medicine and Caring Sciences, Linköping University, Linköping, Sweden

**Keywords:** Adverse drug reaction, Drug-related problem, Healthcare, Pharmaceutical care, Pharmacovigilance, Potentially inappropriate medication

## Abstract

The prevalence of harms in healthcare related to drug treatment is often quantified using terms developed for pharmacovigilance and pharmaceutical care. In this overview, we guide through the definitions and the settings for which they were developed, with the underlying intention to facilitate the interpretation of hitherto available research intended to contribute information regarding the magnitude of the problem in healthcare and to provide guidance for future research. To start, the regulatory/academic definitions of an adverse drug reaction (ADR) and a drug-related problem (DRP) are considerably broader than a literal interpretation would suggest. ADRs are defined for the pharmacovigilance setting, and for drug safety reasons the opposite of the benefit of the doubt rules; if it cannot be excluded that the medication has caused or contributed to an event, it will be a suspected ADR. DRPs represent the pharmaceutical care setting where every aspect is included that could potentially be problematic; a manifested problem is not required. When quantifying the prevalence of harms related to drug treatment in the healthcare setting, however, it may not be considered reasonable to count every circumstance that could possibly be an ADR or everything that could potentially be problematic. Therefore, definitions developed for the pharmacovigilance and the pharmaceutical care settings are not fully applicable to estimate the magnitude of drug treatment problems in healthcare. Proposed guidance for the future includes cautious interpretation of research results, as well as a conscious choice of definitions according to purpose and tempered reporting in research.

## What is the problem?

Drug-related harms in healthcare deserve attention. Indeed, irrespective of the medication being prescribed and used correctly or not, it may imply undesired consequences for the patient. Furthermore, harmful events may have consequences for the use of healthcare resources. In addition to numerous research studies, drug-related harms in healthcare form the basis for overarching initiatives like the Patient Safety flagship from the World Health Organization (WHO) [1] including the WHO policy for medication without harm [2].

When it comes to the quantification of harms in healthcare related to drug treatment, a key publication in the BMJ, studying 18,820 patients >16 years of age admitted to two hospitals in the UK in 2001‒2002, reported that 6.5% of the admissions were caused by adverse drug reactions (ADRs) [3]. Reading more recent research, however, researchers, authorities, and healthcare decision-makers may face considerably higher prevalences. Indeed, prevalence figures from original publications used in meta-analyses with substantial heterogeneity span up to 29% [4] and 41% [5]. Although those two publications are restricted to older patients [6, 7], the figures may be experienced surprisingly large, and thus unreliable, from a healthcare perspective.

High prevalence figures of drug-related admissions, combined with meta-analyses that could be literally interpreted to report that every fourth older patient would be prescribed drugs that are inappropriate [8] and that an older person in general would have three to four problems that have to do with their drug treatment [9, 10], may have implications. Indeed, as a consequence of evidence-based medicine, which has evolved over the last decades [11, 12], systematic reviews have come to constitute a cornerstone to inform decision-making in healthcare. Thus, the inclusion of studies with unreliable figures in meta-analyses implies a risk of overestimated pooled estimates to form the basis for healthcare decisions. Interestingly, most of the above-mentioned systematic reviews advocate healthcare interventions in the abstract conclusion, referring to the magnitude of their result [4, 5, 9, 13], and so do original studies reporting extreme figures regarding the prevalence of drug-related admissions [6, 14].

Pharmacovigilance, as defined by the WHO, represents the science and activities relating to the detection, assessment, understanding, and prevention of adverse effects or any other medicine/vaccine-related problem [15]. “Pharmaceutical care” represents other stakeholders in the drug safety context, being defined by the Pharmaceutical Care Network Europe (PCNE) as “the pharmacist’s contribution to the care of individuals in order to optimize medicines use and improve health outcomes” [16]. A literature review 10 years ago revealed that multiple terms were used within the field of patient safety related to medication, including ADRs, adverse drug events (ADEs), adverse events (AE), medication errors (ME), and drug-related problems (DRP) [17]. In this overview, we present basic definitions developed for the pharmacovigilance and the pharmaceutical care settings and reflect on consequences when they are instead used to quantify the magnitude of the problem regarding drug-related harms in healthcare.

## Definitions and settings

### Adverse drug reactions and adverse events

Definitions of the terms ADR and AE have been developed within the pharmacovigilance setting. An early definition of an ADR originated from the WHO in the 1970s [18] (Table 1). For some decades, the term focused on events occurring when drugs were used at normal doses. This definition of the term corresponded well with the general understanding of the adverse consequences of drug treatment when used as intended in the clinical context. Therefore, it also worked well as a basis for discussions on expected benefits and risks regarding a specific drug treatment for a specific individual during the physician-patient encounter.

**Table 1:** Definition examples

Term	Origin	Definition	Suspected causal relationship drug-event required	Manifested harm required (clinical outcome)	Intent
ADR	WHO original	“An *adverse reaction* to a drug is one that is noxious, is unintended, and occurs at doses normally used in man” [18]	Yes	Yes	Pharmacovigilance
	Expanded definition frequently used	“An appreciably harmful or unpleasant reaction, resulting from an intervention related to the use of a medicinal product, which predicts hazard from future administration and warrants prevention or specific treatment, or alteration of the dosage regimen, or withdrawal of the product” [19]	Yes	Yes	Pharmacovigilance
	EU Pharmacovigilance Directive	“The definition of the term ‘adverse reaction’ should be amended to ensure that it covers noxious and unintended effects resulting not only from the authorised use of a medicinal product at normal doses, but also from medication errors and uses outside the terms of the marketing authorisation, including the misuse and abuse of the medicinal product” [20]	Yes	Yes	Pharmacovigilance
AE	WHO collaborating centres in consensus	“Any untoward medical occurrence in a patient or clinical investigation subject administered a pharmaceutical product and which does not necessarily have to have a causal relationship with this treatment” [21]	No	Yes	Pharmacovigilance
Medication error	ASHP	“A dose of medication that deviates from the physician’s order as written in the patient’s chart or from standard hospital policy and procedures” [22]	NA	No	Pharmaceutical care
	EMA	“A medication error is an unintended failure in the drug treatment process that leads to, or has the potential to lead to, harm to the patient” [23]	NA	No	Pharmacovigilance
DRP	PCNE	”An event or circumstance involving drug therapy that actually or potentially interferes with desired health outcomes” [24]	NA	No	Pharmaceutical care
	Indicators of prescribing quality (PIMs/PPOs) (DRP subset)	“A measurable element of prescribing performance for which there is evidence or consensus that it can be used to assess quality and, hence, be used in changing the quality of care provided” [25]^1^	NA	No	Pharmaceutical care. Suggested in core outcome sets for the evaluation of interventions aimed at improved prescribing [26–28]

*ADR* adverse drug reaction, *AE* adverse event, *ASHP* American Society of Health-System Pharmacists, *DRP* drug-related problem, *EMA* European Medicines Agency, *NA* not applicable, *PCNE* Pharmaceutical Care Network Europe, *PIM* potentially inappropriate medication, *PPO* potential prescribing omission, *WHO* World Health Organization

^1^Adjusted from a general indicator of quality of practice performance defined in 1997 [29]

**Fig. 1:**
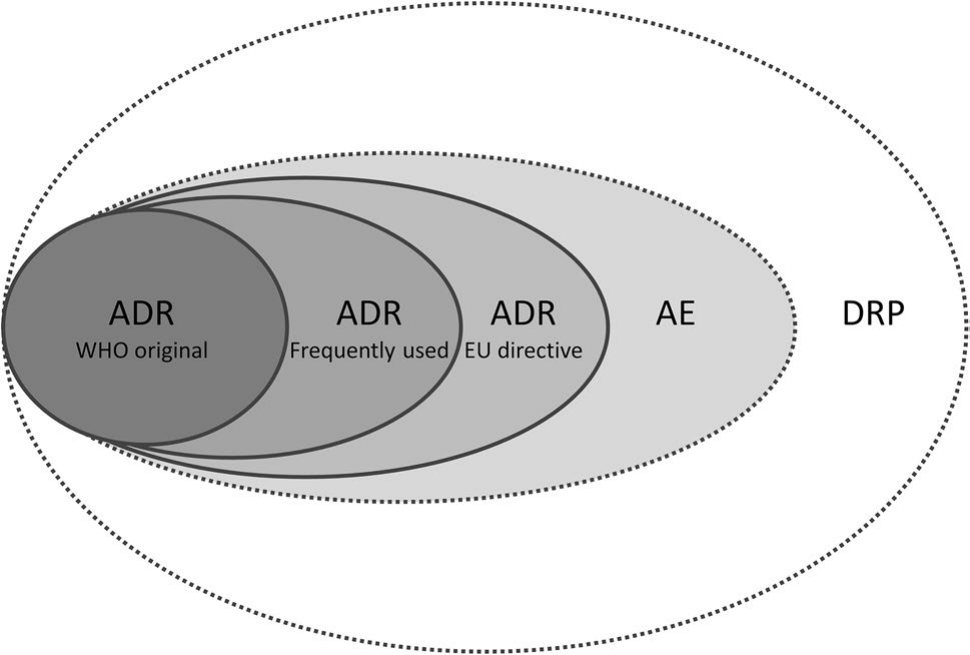
Schematic illustration of the content of terms used in research quantifying the magnitude of the problem regarding harms in healthcare related to drug treatment**.** Shaded areas require an event to have occurred whereas the unshaded area does not require an event. Solid lines represent terms that require at least a possible causal relationship between a drug and an event whereas terms delimited by dashed lines do not require any causality. ADR, adverse drug reaction; AE, adverse event; DRP, drug-related problem; EU, European Union; WHO, World Health Organization

At the turn of the millennium, a new definition of an ADR was proposed within pharmacovigilance. This implied an expanded scope as the explicit restriction regarding the dose was discarded [19] (Figure 1). The impact of the proposed definition is illustrated by the fact that that article has hitherto been cited more than 2000 times [30]. One decade later, the European regulatory definition was revised and expanded the ADR definition further, including events occurring irrespective of the dose used, as well as MEs and misuse or abuse of drugs [20].

In the pharmacovigilance setting, it is natural that the opposite of the benefit of the doubt rules and that all possible negative outcomes of treatment with drugs are identified and reported. Thus, if it cannot be excluded that the medication has caused or contributed to an event, it will be classified as a suspected ADR. Indeed, no event potentially related to a drug must be disregarded in pharmacovigilance. All definitions of an ADR therefore require at least a possible causal relationship between the suspected drug and the event, i.e. that an emerging or worsening disease is not a more likely explanation [19]. “Possible” as a level of causality thus implies that the drug must not be the only, not a necessary, or not even the most probable cause [19]. Thus, spontaneous reporting systems generate safety signals for further evaluation and potential pharmacovigilance actions to protect public health [31].

The AE term is considerably broader than an ADR. It is just an undesired event and does not require a possible causal relationship with a drug—it is independent of any association with an intervention, temporal, or otherwise [21]. The term AE is used in clinical trials [32], and the content can be illustrated by the fact that it also includes events in the placebo group. In the drug safety context, the ADE term must also be mentioned. It has been frequently used [17], in particular by patient safety organisations [33], but has been suggested to be abandoned [34] since an ADE could be either an ADR or an AE and may therefore add to confusion rather than clarification.

### Medication errors and drug-related problems

The terms ME and DRP were developed within the pharmaceutical care setting. An early definition of an ME was made in the 1980s by the American Society of Hospital Pharmacists, focusing on deviations in physicians’ orders and standard policies and procedures [22]. The current definition, used by the European Medicines Agency (EMA), includes all unintended failures in the drug treatment process [23]. The regulatory definition, as well as the broad scope of the current definition of the adjacent DRP term defined by the PCNE, with the stated intent pharmaceutical care, implies that a manifested problem is not required [24, 35]. This, in turn, implies that a causality assessment may not be applicable.

Examples within the first domain of the PCNE definition of a DRP, *drug selection*, are “inappropriate drug according to guidelines/formulary” and “no or incomplete drug treatment in spite of existing indication” [24]. These parts of the domain broadly correspond to potentially inappropriate drugs (PIMs) and potential prescribing omissions (PPOs), for instance, according to the recently updated STOPP/START criteria (Screening Tool of Older Person’s Prescriptions/Screening Tool to Alert to Right Treatment) [36]. PIMs and PPOs reflect what experts consider possibly problematic prescribing [37, 38].

## Implications for quantification of drug-related harms in healthcare

According to our experience, expressions like “adverse drug reaction” and “drug-related problem” are used within the contexts of healthcare and healthcare decision-making without connection to the regulatory/academic definitions applied in pharmacovigilance and pharmaceutical care. Indeed, differences between the understanding of the terms in pharmacovigilance and in patient safety organisations have previously been tabulated (see Table 3 in [33]). Problems arising, when these terms are used to quantify the magnitude of the problem regarding drug-related harms in healthcare, can be illustrated by the quote: “It is suspected that 3% of the population [of Sweden] dies because of a medication error” [39]. This estimate probably represents an extrapolation of the estimation that “almost 3,000 individuals die annually due to a medication error” [in Sweden where almost 100,000 deaths occur every year; translated from Swedish by the authors] presented in the background to, and as one of the rationales for, the Swedish National Pharmaceutical Strategy in 2011 [40]. Reading the underlying study, it appears that 49 (3.1%) out of 1574 consecutive deaths were classified as suspected fatal ADRs from a pharmacovigilance perspective. This definition of an ADR implies, as previously described, a temporal association between the drug and the event, and that it cannot be excluded that the drug treatment could have contributed to some extent. Thus, the words “because” or “due to” within the quote indicate a stronger causal relationship than is actually the case. The Swedish National Pharmaceutical Strategy quote also erroneously equates ADRs with MEs. Finally, their statement may easily be misunderstood in that all MEs would be preventable. In fact, the underlying study showed that merely seven deaths (0.4%) in the study could, to some extent, be considered preventable [41].

As exemplified above, the use of definitions developed for the pharmacovigilance and the pharmaceutical care settings, to quantify the magnitude of the problem regarding harms related to drugs in healthcare, can easily give rise to misunderstandings. Indeed, although drugs should always be included in the process of differential diagnosis, it is important to distinguish between events where it cannot be excluded that the drug treatment could have contributed and events where the drug treatment is the probable reason for the experienced symptoms. Challenges related to these causality assessments have been known for long [42]. One could speculate that physicians, who are medically trained to diagnose patients, may contribute importantly to assessments of the relationship between a drug treatment and an event. This is supported by findings in a meta-research study: between 3.2 and 4.5% of admissions were considered drug-related when physicians contributed to the assessments, i.e. close to the key publication in the BMJ from 2004 [3], whereas 8.8 to 41% of the admissions were considered drug-related when the assessments were performed without medical input [43]. Thus, professional background, i.e. perspective applied, has been reported to account for a great deal of the diversity of the results, whereas heterogeneity of settings and definitions were less conspicuous underlying factors [43].

The fact that DRPs include aspects that do not imply manifested harm or malpractice diminishes its relevance to quantify the prevalence of harms related to drugs in healthcare. Regarding the potential of malpractice, it needs to be noted that the DRP subset of PIMs/PPOs is seldom problematic; merely 7% of detected PIMs/PPOs merited a related medical action according to two experienced physicians in consensus after the circumstances of the specific patient had been considered [44]. Another DRP subset, “Necessary information not provided” within the DRP domain “Dispensing”, further illustrates the inappropriateness of the DRP term to quantify harms in the healthcare setting. This subset includes that it is not clear to the dispensing pharmacist if the prescribing physician was aware of the dispensed drugs and, for instance, the triggered interaction alerts. Such alerts, in turn, seldom merit action as most are already being addressed or are not relevant for the specific patient [45]. Finally, challenges related to the detection of DRPs must be considered. For instance, the inter-rater agreement in detecting PIMs and PPOs is limited; two-thirds have been reported to be discordantly detected, i.e. detected by one assessor but not the other [46].

In summary, definitions that have been developed for the pharmacovigilance and the pharmaceutical care settings will inevitably imply a risk of overestimation of harms related to drugs from a healthcare perspective (Figure 1). Pitfalls related to the definitions of ADRs and DRPs also have implications for research. With a preconception that preventable harmful drug treatment is prevalent, a consequential conclusion may be to target the apprehended underlying problematic prescribing in intervention studies. However, despite great expectations [47, 48], the interventions in two large European randomised controlled trials did not display patient-relevant effects [49, 50]. For one of these, the primary outcome result pointed in the wrong direction [49]. For the other, unrealistic estimates of harms related to drugs, including preventability, may have contributed to unrealistic expectations of possible benefits; the sample size calculations were based on an annual prevalence of drug-related admissions of 20% and an anticipated 30% relative risk reduction. The achieved absolute risk reduction, however, turned out to be 0.5 percentage points (not statistically significant), with a hazard ratio of 0.95 (0.77 to 1.17) [50].

## Suggestions for the future

To estimate the magnitude of the problem regarding patient harms in healthcare caused by the drug treatment, we suggest that, for this purpose, not to use the pharmacovigilance cut-off regarding causality for ADRs, and not to use the pharmaceutical care definition of DRPs (Table 2). Indeed, from a healthcare perspective, it cannot be considered reasonable to count as harms every circumstance that could possibly be an ADR or everything that could potentially be problematic. Instead, we suggest that research on the prevalence of ADR-related patient outcomes should either clearly state the implications of using possible as a cut-off, or, preferably, report assessments of possible and probable ADRs separately. In addition, physicians need to be involved in the assessments to ascertain that differential diagnosis is considered. Furthermore, the original WHO definition, requiring normal doses and not including misuse or MEs, could preferably be applied. When studying all drug-related harms, the consequences for the interpretation in the clinical context of using the latter and wider definition need to be explicit in the conclusion as well as the abstract conclusion.

**Table 2:** Suggestions for quantification of harms related to drugs in the healthcare setting, i.e. to estimate the magnitude of the problem

Suggestions	Underlying reason
Caution is required when definitions developed for the pharmacovigilance and pharmaceutical care settings are used	To avoid overestimated figures
Consider using the original definition of an ADR provided by the WHO, or describe explicitly the consequences of not doing so in the abstract conclusion	To minimise the risk of misinterpretation: In the patient care setting, ADRs will not always be understood as intoxications, misuse, or medication errors
Report ADRs with a possible and probable/certain causal relationship separately	To facilitate meaningful interpretation for the healthcare setting
Report inter-rater agreement	To provide information regarding the reliability
Involve physicians in the ADR assessments	To ascertain that differential diagnosis is considered
Interpret prevalence figures regarding PIMs/PPOs (a subset of DRPs) with caution	They do not correctly reflect the quality of drug treatment
In research studies to evaluate medication correction initiatives, use realistic estimates of drug-related admissions and preventability	To allow reasonable power calculations and to minimise the risk of statistical type II errors
In conclusions, as well as abstract conclusions, avoid a translation of ADR/DRP prevalence results to advocating healthcare interventions	Not to provide recommendations that are not supported by the results

*ADR* adverse drug reaction, *DRP* drug-related problem, *PIM* potentially inappropriate medication, *PPO* potential prescribing omission, *WHO* World Health Organization

In research studies investigating the effects of medication correction initiatives in the healthcare setting, we suggest caution in using DRPs as main outcome measures; in many cases, they do not even reflect the potential of patient harm and thus have limited relevance for healthcare decision-making. Furthermore, we suggest that results regarding the DRP subset PIMs/PPOs should be interpreted with caution. Although PIMs/PPOs have been suggested to be included in core outcome sets in studies evaluating interventions for improved prescribing [26–28], they perform poorly as diagnostic tools to identify inadequate drug treatment [51]. In fact, they have been reported to be comparable with a simple count of the number of drugs in the medication list [51], which, in turn, has been associated with the burden of disease [52].

As mentioned in the introduction to this article, the cited systematic reviews with focus on ADR/DRP-related hospital admissions advocate healthcare interventions in the abstract conclusion, referring to the magnitude of their results [4, 5, 13]. The same applies to the systematic review regarding the prevalence of DRPs [9], and the study reporting extreme figures regarding the prevalence of drug-related admissions [6]. Thus, the authors explicitly translate their results regarding the magnitude of the prevalence of ADRs/DRPs to healthcare decision-making, that is, equals results regarding terms developed for other settings to healthcare, and implicitly suggest that such prevalence studies constitute an appropriate evidence base for interventional studies. As aforesaid conclusions are not supported by the results, however, we suggest that such translations should be avoided or, at least, be accompanied by an explicit statement about the limitations of such a translation, in particular in abstract conclusions that ought to present the key content of an article.

In conclusion, the current overview illustrates how insights in drug safety term definitions, including the settings for which they were developed, are essential. Using the suggested guidance in interpretation of research results for healthcare decision-making, as well as in the performance and reporting of studies intended to contribute evidence for such decisions, may contribute to evidence-based medicine. Indeed, initiatives for patient safety and minimising the risk of drug-related harms deserve attention in healthcare.

## Data Availability

No datasets were generated or analysed during the current study.
